# A Comprehensive Review of Advancements in Diagnostic Imaging: Unveiling Oral Cavity Malignancies Using Computed Tomography

**DOI:** 10.7759/cureus.64045

**Published:** 2024-07-07

**Authors:** Paschyanti R Kasat, Pratapsingh Parihar, Shivali V Kashikar, Pratiksha Sachani, Priyal Shrivastava, Utkarsh Pradeep, Smruti A Mapari, Gautam N Bedi

**Affiliations:** 1 Radiodiagnosis, Jawaharlal Nehru Medical College, Datta Meghe Institute of Higher Education and Research, Wardha, IND; 2 Medicine, Jawaharlal Nehru Medical College, Datta Meghe Institute of Higher Education and Research, Wardha, IND; 3 Obstetrics and Gynecology, Jawaharlal Nehru Medical College, wardha, IND

**Keywords:** technological advancements, tumor staging, diagnostic imaging, early detection, computed tomography (ct), oral cavity malignancies

## Abstract

Early detection of oral cavity malignancies is essential for improving treatment outcomes and patient survival rates. Diagnostic imaging, particularly computed tomography (CT), plays a pivotal role in the early identification and detailed assessment of these malignancies. This comprehensive review explores the advancements in CT imaging and its application in diagnosing oral cavity cancers. It discusses the anatomy and physiology of the oral cavity, the clinical characteristics of common malignancies, and the principles and protocols of CT imaging. The review highlights the diagnostic features of oral malignancies on CT, including distinguishing benign from malignant lesions and staging criteria. Emerging technologies, such as higher-resolution imaging, integration with other modalities, and the potential of artificial intelligence, are examined for their role in enhancing diagnostic accuracy. The clinical implications, challenges, and future directions in the use of CT imaging for oral cavity malignancies are also discussed. This review underscores the importance of continued research and technological advancements in optimizing the use of CT for early detection and effective management of oral cavity cancers.

## Introduction and background

Early detection of oral cavity malignancies is crucial for improving patient outcomes and survival rates. Oral cancers, including those of the lips, tongue, floor of the mouth, and other oral cavity structures, often present with nonspecific symptoms in their early stages, leading to delayed diagnosis and treatment initiation [1]. However, when detected early, the chances of successful treatment and cure are significantly higher. Thus, highlighting the importance of strategies aimed at early detection and diagnosis of these malignancies is paramount [2]. Diagnostic imaging plays a pivotal role in the early detection of oral cavity malignancies. Traditional clinical examination techniques may not always be sufficient to identify subtle or deep-seated lesions. Imaging modalities such as computed tomography (CT) offer a non-invasive means to visualize the internal structures of the oral cavity with high detail and accuracy. By providing valuable insights into the extent and characteristics of lesions, imaging aids in the timely identification, staging, and management planning of oral cavity malignancies [3].

CT is a widely utilized imaging modality in the evaluation of oral cavity lesions. It utilizes X-rays to generate cross-sectional images of the body, allowing for detailed visualization of anatomical structures and pathological abnormalities [4]. CT imaging provides information regarding the size, location, morphology, and tissue characteristics of oral cavity lesions, aiding clinicians in making accurate diagnoses and treatment decisions. Its ability to capture three-dimensional images and detect subtle changes makes it an invaluable tool in the comprehensive assessment of oral cavity malignancies [4]. The purpose of this review is to provide a comprehensive overview of the role of CT in the early detection and diagnosis of oral cavity malignancies. By examining the current literature and discussing key concepts, imaging techniques, diagnostic features, and emerging trends, this review aims to elucidate the importance of CT imaging in the management of oral cavity lesions. Additionally, it seeks to highlight the clinical significance, challenges, and future directions in the use of CT for optimizing patient care in the context of oral cavity malignancies.

## Review

Oral cavity malignancies: Types and characteristics

Common Types of Oral Cavity Malignancies

**Figure 1:**
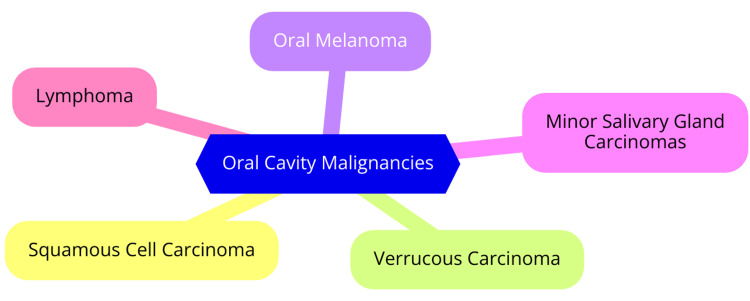
Common types of oral cavity malignancies Image Credit: Dr. Paschyanti R. Kasat

Oral cavity malignancies encompass various cancers that develop in the mouth and throat regions. Among these, squamous cell carcinoma stands as the most prevalent type, constituting approximately 90% of all oral cavity cancers. Derived from the flat, scale-like squamous cells lining the oral mucosa, this carcinoma can be categorized into distinct subtypes based on cellular composition and anatomical location within the oral cavity [5]. Additionally, less common forms of oral cavity cancers include verrucous carcinoma, a rare subtype of squamous cell carcinoma characterized by slow growth and infrequent metastasis; oral melanoma, a skin cancer originating in melanocytes, the cells responsible for skin pigmentation; minor salivary gland carcinomas, comprising various subtypes of oral cancer originating from the salivary glands dispersed throughout the oral and pharyngeal mucosa; and lymphoma, cancer affecting lymphoid tissues situated at the base of the tongue or within the tonsils [6]. Each variant of oral cavity cancer presents distinctive characteristics and symptoms. Lip cancer typically manifests with symptoms such as persistent lip pain or numbness, non-healing sores, a lump, a discolored patch, or a thickened area on the lip. Symptoms of tongue cancer often involve mouth numbness, chronic sore throat, painful swallowing, or the appearance of discolored patches on the tongue. Throat cancer symptoms may include difficulty or discomfort during swallowing, persistent sore throat, unexplained weight loss, or coughing up blood [7]. Diagnosing oral cavity cancer entails a comprehensive approach involving clinical examination, imaging modalities such as CT and MRI, and biopsy to confirm the presence and extent of the disease. Treatment strategies vary depending on the type and stage of the cancer but commonly include surgical intervention, radiation therapy, and chemotherapy [8]. Common types of oral cavity malignancies are shown in Figure 1.

Clinical Characteristics and Presentations

The clinical characteristics and presentations of oral cavity malignancies encompass a spectrum of symptoms and signs. Squamous cell carcinoma, constituting over 90% of oral cavity cancers, typically manifests as erythroplakia (redness), erythroleukoplakia (combined white and red lesion), indurated (hard) areas of ulceration, non-healing ulcers or sores, or palpable lumps or thickening. These manifestations can be subtle and may not readily manifest, underscoring the importance of routine dental check-ups and oral cancer screenings for early detection [9]. Early symptoms of oral cavity malignancies often include persistent soreness or discomfort, white, red, or speckled patches, non-healing ulcers or sores, or palpable lumps or thickening. Given their varied nature, these symptoms may overlap with those of other conditions, necessitating consultation with a healthcare professional for accurate diagnosis. Conversely, later symptoms are typically more pronounced and may encompass mucosal ulceration, denture-related swelling, impaired tongue or jaw mobility, difficulties in chewing or swallowing, tongue or mouth numbness, or palpable neck or jaw lumps [10]. Verrucous carcinoma, a rare subtype of oral cavity cancer, typically presents as a cauliflower-like mass, often observed on the mandibular gingiva and buccal sulcus. Characterized by its slow growth rate and low metastatic potential, this subtype frequently manifests with lymphadenopathy and palpable lymph nodes in the submandibular and deep cervical regions, indicating advanced disease [11]. The tongue and floor of the mouth serve as primary sites for oral cavity malignancies, accounting for nearly half of all cases. Pain is the predominant presenting symptom, particularly in tongue cancers, due to their mobile and sensitive nature. Early lesions may be asymptomatic and incidentally discovered, whereas advanced cancers commonly elicit pain. Timely diagnosis hinges on comprehensive clinical history and examination, as early indicators may be subtle and easily overlooked. Routine dental check-ups and oral cancer screenings are indispensable for promptly detecting and effectively managing oral cavity malignancies [12].

Importance of Accurate Diagnosis and Staging

Accurate diagnosis and staging of oral cavity malignancies are paramount for tailoring appropriate treatment strategies and predicting patient outcomes [13-16]. The predominant staging system utilized is the American Joint Committee on Cancer (AJCC) TNM system, which evaluates the primary tumor (T), regional lymph nodes (N), and distant metastasis (M) [16]. A comprehensive staging process relies on meticulous clinical examination and imaging studies such as CT and MRI [13-16]. CT is the preferred modality for diagnosing primary oral cavity lesions (excluding those of the oral tongue and hard palate). At the same time, MRI is favored for lesions of the oral tongue and floor of the mouth [14]. Additionally, 18-fluoro-deoxyglucose positron emission tomography (FDG-PET) CT may be employed for diagnosis in specific contexts [14]. Imaging is pivotal in delineating disease extent, identifying regional and distant metastases, and formulating tailored treatment plans [13-16]. Treatment approaches vary depending on the stage of the disease. Early-stage oral cancers can often be effectively managed with surgery alone, whereas advanced cancers typically necessitate a combination of surgical intervention, radiation therapy, and/or chemotherapy [13,15]. Since treatment decisions are frequently based on clinical and radiological staging, precise staging is imperative [15]. Understaging may lead to inadequate treatment, whereas overstaging could result in unnecessary morbidity stemming from aggressive therapeutic interventions [15]. Therefore, achieving accurate diagnosis and staging through a comprehensive blend of clinical assessment and imaging techniques is indispensable for optimizing treatment efficacy and enhancing patient outcomes in oral cavity malignancies.

CT in oral cavity imaging

Principles of CT Imaging

CT imaging is rooted in fundamental physical principles, technical intricacies, and clinical applications essential for its role in medical diagnostics. It harnesses X-ray technology and sophisticated computer algorithms to produce detailed cross-sectional images of the body, offering comprehensive 3D representations with remarkable clarity [17,18]. Regarding physical principles, CT imaging operates based on differential attenuation, where variations in X-ray attenuation by absorption or scattering in different tissues yield differences in image intensity. Soft-tissue contrast in CT predominantly arises from disparities in physical density, enabling the visualization of subtle density variations through image reconstruction and windowing techniques [19]. From a technical perspective, CT scanners function by emitting X-rays and utilizing detectors to measure the attenuation of X-rays as they traverse through the body. The data collected during the scanning process is then meticulously processed using computer algorithms to generate cross-sectional images, furnishing detailed insights into tissue density and structure [17]. Clinically, CT scans serve as invaluable tools for diagnosing and managing diverse medical conditions. They offer high-resolution, cross-sectional images that facilitate precise visualization, aiding clinicians in making accurate assessments and treatment decisions. Moreover, CT imaging procedures are versatile, rapid, and non-invasive, with the option of employing contrast agents to enhance visibility and delineate anatomical structures [19]. The quality of CT images is contingent upon various factors, including image contrast, spatial resolution, image noise, and artifacts. Ensuring optimal image quality while minimizing radiation exposure necessitates adherence to proper clinical protocols. This involves meticulous consideration of parameters such as kilovolt peaks, amperages, slice thicknesses, and reconstruction filters to achieve diagnostically acceptable image quality at optimized radiation doses [17].

Advantages and Limitations in Imaging Oral Cavity Structures

CT and MRI offer faster image acquisition than MRI, making them more suitable for patients with limited time or those who may have difficulty holding still for extended periods. Moreover, CT is particularly valuable for assessing cortical bone involvement, crucial for staging and treatment planning in oral cavity malignancies. Multidetector CT (MDCT) can mitigate patient movement artifacts by capturing images in a single short breath hold or during normal breathing [20]. Conversely, MRI provides superior soft-tissue resolution, especially beneficial for staging oral cavity malignancies involving the floor of the mouth and complex disease processes. Additionally, MRI is adept at delineating marrow involvement and perineural spread of tumors, vital for precise staging and treatment planning. However, MRI is susceptible to artifacts induced by swallowing-related movements, potentially compromising its diagnostic accuracy [21]. The puffed cheek technique in CT proves beneficial for evaluating mucosal tumors where mucosal surfaces are opposed, facilitating better visualization of small tumors that may escape detection on radiographs. Angled gantry CT can mitigate dental amalgam artifacts by imaging along the plane of the mandible, thereby enhancing visualization of oral cavity structures. Oral ultrasound delivers high-resolution imaging for assessing salivary duct or gland pathology and tongue tumor thickness, a significant independent prognostic factor for nodal metastasis and overall survival [22]. Despite their advantages, both CT and MRI have limitations. Dental amalgam artifacts in CT scans can obscure oral cavity details, necessitating additional scans with angled gantry CT to diminish these artifacts. Moreover, CT and MRI scans can be costly and inaccessible in all regions, limiting their widespread utilization. Additionally, both modalities are susceptible to motion artifacts, which can degrade image quality and compromise diagnostic accuracy. MRI is also contraindicated in patients with cardiac pacemakers and may induce claustrophobia, restricting its use in certain patient populations [23].

Protocols and Techniques for Optimal Oral Cavity Imaging

To optimize oral cavity imaging, a helical scanner with a thin slice thickness of 0.75 mm is recommended. Additionally, administering iodine-based intravenous contrast at a flow rate of 3-5 mL/sec with a total volume of 80 mL enhances image clarity. Implementing the "puffed cheek" technique, where the patient blows uniformly through pursed lips while breathing normally, helps to separate the buccal and gingival surfaces. Image acquisition should occur over 40-50 seconds, with reconstructed images obtained using soft tissue and bone algorithms. It's also advisable to obtain coronal and sagittal reformatted images in addition to axial images [24]. Dental amalgams can induce significant artifacts in CT scans. To mitigate this issue, additional scans can be conducted through the affected region with gantry angulation along the plane of the mandible. This technique effectively reduces dental amalgam artifacts and enhances the visualization of oral cavity structures [25]. CT proves particularly beneficial for staging oral cavity malignancies as it allows for assessing tumor extent, depth of invasion, and nodal involvement. Conversely, MRI is well-suited for staging oral cavity malignancies affecting the floor of the mouth and involving complex disease processes spanning multiple anatomical spaces. MRI also excels in depicting marrow involvement and perineural spread of tumors [26]. Intraoral ultrasound is valuable for evaluating salivary duct or gland pathology and measuring tongue tumor thickness. Tumor thickness exceeding 4 mm on ultrasound is an independent prognostic indicator for nodal metastasis and overall survival. This technique offers high-resolution imaging using a small-footprint intra-oral probe, complementing CT and MRI in diagnosing and staging oral cavity malignancies [27].

Diagnostic features of oral cavity malignancies on CT

Radiological Features of Benign vs. Malignant Lesions

Distinct radiological features aid in the diagnosis of benign lesions of the mandible. Cystic lesions, such as periapical (radicular) cysts, follicular (dentigerous) cysts, and odontogenic keratocysts, typically present as unilocular or multilocular lucent lesions with smooth borders. Often associated with an impacted tooth, these lesions can manifest in various mandibular locations. Solid benign tumors, including ameloblastomas, odontomas, ossifying fibromas, and periapical cemental dysplasia, appear as multilocular or unilocular solid lesions with varying degrees of bone destruction, commonly observed in periapical regions and often linked with an impacted tooth [28]. Conversely, malignant mandible lesions, such as squamous cell carcinomas, osteosarcomas, and metastatic tumors, exhibit radiological features distinct from benign lesions. Squamous cell carcinomas typically manifest as multilocular or unilocular solid lesions with significant bone destruction and invasion of surrounding tissues. Similarly, osteosarcomas and metastatic tumors present as multilocular or unilocular solid lesions with substantial bone destruction and invasion of adjacent tissues, typically affecting older individuals and occurring anywhere within the mandible [29,30].

Several key radiological features are crucial for distinguishing benign from malignant lesions. The contour of the lesion border plays a pivotal role, with benign lesions often characterized by smooth borders and malignant lesions displaying irregular or ill-defined borders. Additionally, the lesion's location within the mandible is pertinent, as benign lesions tend to localize in specific regions like periapical areas, whereas malignant lesions can arise anywhere within the mandible. Furthermore, the age of onset of a lesion holds significance, with benign lesions typically emerging at a younger age and malignant lesions manifesting at an older age [31]. Understanding the radiological features of lesions is imperative for accurate diagnosis and effective treatment planning. These features aid in differentiating between benign and malignant lesions, informing treatment decisions and patient management. For instance, while benign lesions may be managed conservatively with approaches like root canal therapy or extraction, malignant lesions typically necessitate more aggressive interventions such as surgery and radiation therapy. Therefore, a comprehensive grasp of the radiological features of mandibular lesions is vital for delivering optimal care to affected patients [31].

Tumor Staging Criteria Based on CT Findings

Tumor staging criteria based on CT findings entail thoroughly evaluating tumor size and extent, nodal involvement, and the presence of distant metastases. The T stage, which focuses on tumor size and extent, typically comprises four stages: T0 indicating no evidence of a tumor, T1 denoting a small tumor, T2 representing a larger tumor, and T3 indicating a large tumor. The most advanced stage, T4, is characterized by a very large tumor [32]. Concurrently, the N stage, assessing nodal involvement, consists of four stages: N0 indicating no cancer in lymph nodes, N1 representing a small amount of cancer in lymph nodes, N2 indicating a moderate amount, and N3 denoting an extensive amount. This staging system aids in delineating the extent of nodal involvement, crucial for treatment planning and predicting patient outcomes [32]. Furthermore, the M stage evaluates the presence of distant metastases and is categorized into two stages: M0, indicating no detectable metastasis, and M1, representing cancer spread to other body parts. This staging framework offers a comprehensive grasp of disease extent, facilitating the development of effective treatment strategies and monitoring disease progression [32]. The TNM method, amalgamating T, N, and M stages, is widely adopted for cancer staging based on CT findings. This standardized approach aids in treatment planning, prognostication, and the advancement of novel therapies for cancer patients. By furnishing a clear and consistent framework for assessing the cancer stage, the TNM method plays a pivotal role in enhancing cancer diagnosis and management [32].

Case Studies Illustrating Diagnostic Challenges and Solutions

In one case, a patient initially presented with symptoms resembling a common cold, but further testing revealed an underlying rare, yet treatable viral strain. The pivotal factor was the utilization of a highly specific molecular assay capable of detecting the elusive pathogen amidst the benign microbial flora. Ensuring proper sample handling and maintaining a meticulous chain of custody was also imperative to preserve sample integrity [33,34]. In another instance, a woman with a breast mass identified during a physical examination received an initial diagnostic mammogram interpretation indicating it was "probably benign". However, the mass had significantly increased upon the patient's return five months later. Delays in scheduling the biopsy resulted in a seven-month delay in diagnosing invasive breast cancer. This case underscores the importance of correlating imaging findings with physical examination outcomes and emphasizes not relying solely on one diagnostic modality [35]. Similarly, an 18-year-old man with untreated depression presented with symptoms of paroxysmal supraventricular tachycardia (PSVT). The diagnostic challenge stemmed from gender bias, leading to the dismissal of PSVT in young men as anxiety. Strategies to mitigate diagnostic uncertainty include maintaining a high index of suspicion, obtaining an electrocardiogram (ECG) during symptomatic episodes, and considering provocative testing [35]. Lastly, a woman endured years of symptoms resembling woozy spells after gym sessions before receiving a diagnosis of endometriosis. Endometriosis often faces missed or delayed diagnosis due to a combination of patient, provider, and healthcare system factors. Increased awareness and a willingness to thoroughly investigate persistent symptoms are crucial to improve early detection and management [35].

Emerging technologies and future directions

Innovations in CT Technology for Oral Cavity Imaging

Multi-slice computed tomography (MSCT) scanners have multiple detectors that concurrently acquire tomographic data at various slice locations. This innovation substantially reduces scan duration and radiation exposure compared to earlier incremental scanning methods [36]. Dual-energy computed tomography (DECT) represents a recent advancement utilizing two different energy levels to generate images. While DECT holds promise for studying oral cavity diseases, its routine application is currently hindered by the limited availability of DECT scanners [37]. Cone-beam computed tomography (CBCT) is extensively used in dentistry due to its remarkable precision and accessibility within dental practices. Offering detailed images of the oral cavity and adjacent structures, CBCT serves as a valuable tool in diagnosing and managing various oral diseases [38,39]. Spiral computed tomography (spiral CT) scanners, introduced in the late 1980s, facilitate continuous patient movement through the rotating gantry, thereby reducing exposure time and radiation dose compared to incremental scanners [36]. Integrating CT and MRI modalities presents a synergistic approach to evaluating oral cavity pathologies. CT assesses cortical bone involvement, while MRI boasts superior soft-tissue resolution. Particularly beneficial for staging oral cavity malignancies involving the floor of the mouth and complex disease processes, this integrated approach harnesses the complementary strengths of each modality [40].

Integration of CT With Other Imaging Modalities

The integration of CT with other imaging modalities, known as multimodality imaging, represents a significant advancement in diagnostic capabilities within the medical field. This integration entails combining CT with modalities such as PET, SPECT, and MRI to furnish comprehensive imaging data, thereby enhancing diagnosis and treatment planning [41,42]. Specifically, multimodality imaging, notably the fusion of PET/CT and SPECT/CT, has transformed medical imaging by providing inherently matched images from both modalities within a single study. This integration facilitates superior anatomical and functional imaging, more precise assessments, and improved patient outcomes [42]. The emergence of hybrid imaging systems, exemplified by PET/CT and SPECT/CT, has obviated the need for retrospective software registration of images. Instead, these systems offer seamless integration of functional and anatomical data, thereby enhancing spatial and temporal registration and bolstering the accuracy of diagnostic information across various medical conditions [41,42]. The amalgamation of CT with other imaging modalities through multimodality imaging has markedly bolstered medical diagnostic capabilities. This approach allows for a comprehensive evaluation of patients and enables the formulation of precise treatment strategies grounded in combined anatomical and functional information [42].

Potential Role of Artificial Intelligence (AI) in Enhancing Diagnostic Accuracy

In medical imaging analysis, AI algorithms play a pivotal role in scrutinizing medical images like X-rays, MRIs, and CT scans, thereby enhancing the detection of abnormalities and the accuracy of disease diagnoses. Deep learning techniques, in particular, have exhibited proficiency in expert-level disease identification across various medical imaging settings [43,44]. Moreover, AI can analyze tissue samples with heightened precision in pathology compared to traditional methods. This advancement not only aids in diagnosing various cancer types but also streamlines workflow processes within pathology labs [43]. AI's predictive diagnostics capability leverages patient data, encompassing medical history, symptoms, and laboratory results, to forecast the likelihood of disease occurrence. This predictive modeling empowers clinicians to make more informed decisions, mitigating the risk of misdiagnosis [45]. Integrating AI into virtual primary care settings also bolsters patient intake processes and diagnostic recommendations. Studies indicate high diagnostic accuracy rates in these settings, with AI achieving 90% or higher agreement rates with clinicians [46]. AI systems rapidly and accurately process vast datasets, encompassing medical records, imaging data, laboratory results, and genetic information. This proficiency enables more precise diagnoses and facilitates the formulation of personalized treatment plans tailored to individual patient needs [43]. Furthermore, AI streamlines healthcare workflows by automating routine tasks, thereby liberating healthcare professionals from wasting more time to direct patient care and intricate clinical tasks. This operational efficiency enhancement improves overall diagnostic accuracy and patient management [43]. By harnessing enhanced pattern recognition capabilities, AI algorithms can discern intricate patterns within medical data that might elude human observation, culminating in more accurate diagnoses and reduced errors [43]. Moreover, AI serves as a support tool for clinicians, furnishing evidence-based recommendations and insights that inform informed decision-making processes and developing tailored treatment strategies [43].

## Conclusions

In conclusion, CT plays an indispensable role in the early detection and diagnosis of oral cavity malignancies, offering detailed anatomical visualization and the ability to distinguish between benign and malignant lesions. This imaging modality significantly enhances clinical assessment and treatment planning through precise tumor staging and characterization. Continued advancements in CT technology, including higher resolution imaging and integration with other modalities, alongside the incorporation of AI, promise to further improve diagnostic accuracy and efficiency. Ongoing research and technological innovations are essential for optimizing the use of CT in clinical practice, ultimately leading to better patient outcomes and higher survival rates. The future of CT imaging in the context of oral cavity malignancies is bright, with its evolving capabilities poised to make substantial contributions to early detection and effective management of these cancers.
